# Statistical analysis plan for the POLAR-RCT: The Prophylactic hypOthermia trial to Lessen trAumatic bRain injury-Randomised Controlled Trial

**DOI:** 10.1186/s13063-018-2610-y

**Published:** 2018-04-27

**Authors:** Jeffrey Presneill, Dashiell Gantner, Alistair Nichol, Colin McArthur, Andrew Forbes, Jessica Kasza, Tony Trapani, Lynnette Murray, Stephen Bernard, Peter Cameron, Gilles Capellier, Olivier Huet, Lynette Newby, Stephen Rashford, Jeffrey V. Rosenfeld, Tony Smith, Michael Stephenson, Dinesh Varma, Shirley Vallance, Tony Walker, Steve Webb, D. James Cooper, On behalf of the POLAR investigators and the ANZICS Clinical Trials Group

**Affiliations:** 1 Australian and New Zealand Intensive Care Research Centre, Monash University, School of Public Health and Preventive Medicine, 99 Commercial Road, Melbourne, 3004 Australia; 2 Department of Intensive Care, The Alfred, Melbourne, Australia; 3 Department of Anaesthesia and Intensive Care Medicine, St Vincent’s University Hospital, Dublin, Ireland; 4 School of Medicine and Medical Sciences, University College, Dublin, Ireland; 5 Centre of Excellence in Traumatic Brain Injury Research, The Alfred, Monash University, Melbourne, Australia; 6 Intensive Care Unit, Royal Melbourne Hospital, Melbourne, Australia; 7 Emergency Medicine, Hamad Medical Corporation, Doha, Qatar; 8 St John New Zealand, Auckland, New Zealand; 9 Department of Radiology, The Alfred Hospital, Melbourne, Australia; 10 Department of Surgery, Monash University, Melbourne, Australia; 11 Intensive Care Unit, Royal Perth Hospital, Perth, Australia; 12 Department of Neurosurgery, The Alfred Hospital, Melbourne, Australia; 13 Ambulance Victoria, Melbourne, Australia; 14 Department of Critical Care Medicine, Auckland City Hospital, Auckland, New Zealand; 15 Réanimation médicale CHRU Jean Minjoz, Besançon, France; 16 Université de Franche – Comte, 1 Rue Claude Goudimel, Besançon, 25030 France; 17 Queensland Ambulance Service, Brisbane, Australia; 18 Department of Surgery, F. Edward Hébert School of Medicine, Uniformed Services University of The Health Sciences (USUHS), Bethesda, MD USA; 19 Emergency and Trauma Centre, The Alfred Hospital, Melbourne, Australia; 20 School of Public Health and Preventive Medicine, Monash University, Melbourne, Australia; 21 Department of Medicine, University of Melbourne, Melbourne, Australia; 22 Department of Anaesthesia and Intensive Care Medicine, Hôpital de La Cavale Blanche, CHRU de Brest, Brest, France; 23 UFR de médecine et des sciences de la santé, Université de Bretagne Occidental, Brest, France

**Keywords:** Traumatic brain injury, Cooling, Hypothermia, Outcome, Critical care, Randomised controlled trials

## Abstract

**Background:**

The Prophylactic hypOthermia to Lessen trAumatic bRain injury-Randomised Controlled Trial (POLAR-RCT) will evaluate whether early and sustained prophylactic hypothermia delivered to patients with severe traumatic brain injury improves patient-centred outcomes.

**Methods:**

The POLAR-RCT is a multicentre, randomised, parallel group, phase III trial of early, prophylactic cooling in critically ill patients with severe traumatic brain injury, conducted in Australia, New Zealand, France, Switzerland, Saudi Arabia and Qatar. A total of 511 patients aged 18–60 years have been enrolled with severe acute traumatic brain injury.

The trial intervention of early and sustained prophylactic hypothermia to 33 °C for 72 h will be compared to standard normothermia maintained at a core temperature of 37 °C.

The primary outcome is the proportion of favourable neurological outcomes, comprising good recovery or moderate disability, observed at six months following randomisation utilising a midpoint dichotomisation of the Extended Glasgow Outcome Scale (GOSE). Secondary outcomes, also assessed at six months following randomisation, include the probability of an equal or greater GOSE level, mortality, the proportions of patients with haemorrhage or infection, as well as assessment of quality of life and health economic outcomes. The planned sample size will allow 80% power to detect a 30% relative risk increase from 50% to 65% (equivalent to a 15% absolute risk increase) in favourable neurological outcome at a two-sided alpha of 0.05.

**Discussion:**

Consistent with international guidelines, a detailed and prospective analysis plan has been developed for the POLAR-RCT. This plan specifies the statistical models for evaluation of primary and secondary outcomes, as well as defining covariates for adjusted analyses and methods for exploratory analyses. Application of this statistical analysis plan to the forthcoming POLAR-RCT trial will facilitate unbiased analyses of these important clinical data.

**Trial registration:**

ClinicalTrials.gov, NCT00987688 (first posted 1 October 2009); Australian New Zealand Clinical Trials Registry, ACTRN12609000764235. Registered on 3 September 2009.

**Electronic supplementary material:**

The online version of this article (10.1186/s13063-018-2610-y) contains supplementary material, which is available to authorized users.

## Background

Traumatic brain injury (TBI) is a leading cause of mortality and long-term disability, particularly affecting young people. Even a small increase in the number of TBI victims who are able to live independently, instead of being permanently disabled, would yield major human and economic benefits [1, 2].

The application of *early prophylactic* hypothermia [3] involves the rapid reduction after injury of core body temperature to 33 °C. This therapy has shown promise as an intervention to attenuate TBI [4], with the distinctly early cooling intervention design of the Prophylactic hypOthermia to Lessen trAumatic bRain injury-Randomised Controlled Trial (POLAR-RCT; randomisation within 3 h of estimated time of injury) contrasting with a recent unsuccessful cooling intervention (*therapeutic* hypothermia) applied mostly 12 h or more after TBI [5, 6]. POLAR-RCT is an international, multicentre, randomised, parallel group phase III superiority trial of prophylactic hypothermia delivered to adult critically ill patients with acute severe TBI (Glasgow Coma Score [GCS] < 9) [7]. Despite a recent meta-analysis [8] suggesting improved neurological outcomes from TBI associated with therapeutic hypothermia to 33 °C for 72 h, substantial clinical uncertainty remains in this field [9]. POLAR-RCT aims to evaluate six-month survival and neurological function in adult patients with severe TBI randomised to early prophylactic hypothermia to 33 °C, compared to normothermia.

## Methods/Design

### Study design and definitions

The POLAR-RCT is a multicentre, prospective, two parallel groups, randomised phase III superiority trial evaluating the safety and efficacy of early prophylactic hypothermia targeting a core temperature of 33 °C in adult ICU patients with severe TBI using clinically available refrigerated vests, wraps, jackets, or blankets [7]. Control patients received protocol-directed temperature adjustment, using similar equipment if necessary, to maintain normothermia, defined as a core temperature of 36.5–37.5 °C. Patients enrolled in both the hypothermia and normothermia groups have been managed according to current international evidence-based guidelines [10]. Patient randomisation completed on 10 November 2017 with a total of 511 participants and final collection of all six-month outcome data is anticipated by June 2018.

### Treatment masking (blinding)

Patient temperature is a key clinical vital sign and therefore it is not possible to blind clinical staff to treatment allocation. Bias will be minimised by concealed treatment allocation before randomisation, by protocolised treatment in both groups [7] and by assessment of the primary outcome by centralised, blinded and trained research staff (as accomplished successfully in recent studies including SAFE [11], SAFE-TBI [12], HTS [13] and ATBIS [2]). The primary outcome measure (neurological outcome at six months from randomisation, see below) is subject to minimal ascertainment bias.

### Compliance with good clinical practice

The trial is being conducted, and accumulating data monitored, according to the standard requirements of Good Clinical Practice [14]. Data are collected by trained staff at each study site and entered into a secure, password-protected, encrypted web-based data collection form. Queries of potential inconsistencies are generated automatically by the trial website to facilitate early resolution. Data are stored in a secure server operated by Monash University; data management will be performed by the Clinical Informatics and Data Management Unit, Monash University, Melbourne, Australia [15]. Site monitoring will be performed at each participating hospital by the Trial Project Manager to ensure the study is conducted according to the protocol and all applicable regulations, and to perform source data verification (Additional file 1).

**Fig. 1:**
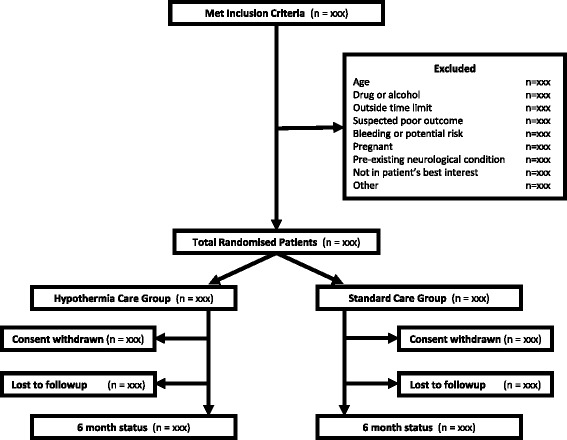
Consort *diagram*

**Fig. 2:**
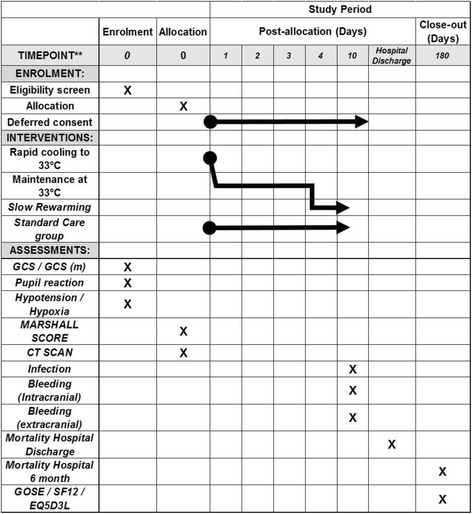
SPIRIT *figure*

General confidence in the final results and conclusions of clinical trials is enhanced when the statistical approaches to outcome analyses are specified before the availability of trial data. The following statistical analysis plan complies with recommendations for the Consolidated Standards of Reporting Trials (CONSORT) (Fig. 1) [16, 17], the Standard Protocol Items: Recommendations for Interventional Trials (SPIRIT) (Fig. 2) [18, 19] (and checklist as an Additional file 2) as well as guidance from the International Conference on Harmonisation of Technical Requirements for Registration of Pharmaceuticals for Human Use, especially ‘Statistical principles for clinical trials E9’ [20] and ‘Structure and content of clinical study reports E3’ [21].

This statistical analysis plan identifies the procedures to be applied to the primary and secondary outcome analyses once trial data validation is complete. Covariates for adjusted analyses and selected subgroups of interest are also pre-specified. This plan defines the intention-to-treat (ITT) full analysis set, as well as exploratory analyses in ‘as-treated’ and ‘per-protocol’ subsets, accompanied by an analysis seeking to estimate the average causal effect of cooling in the presence of non-compliance with the cooling intervention [22].

### Trial population and eligibility

A total of 511 severe TBI patients have been enrolled in Australia, New Zealand, France, Switzerland, Saudi Arabia and Qatar. This 511-patient full analysis set will not include the initial eight patients who were treated within a pre-trial run-in phase. Fourteen emergency departments and five pre-hospital agencies were involved. Patients may have been enrolled in the pre-hospital setting, if specifically trained and qualified staff are available, or by medical staff in the emergency department of participating hospitals. The inclusion and exclusion criteria are presented in Table 1. Eligible patients were randomised to receive prophylactic hypothermia (33 °C for 72 h) or TBI care that is standard (for that hospital) (Figs. 1 and 2).

**Table 1:** Inclusion and exclusion criteria

Pre-hospital inclusion criteria	Emergency Department inclusion criteria
• Blunt trauma with clinical diagnosis of severe TBI and GCS < 9 • Estimated age ≥ 18 and < 60 years • The patient is intubated or intubation is imminent	• Blunt trauma with clinical diagnosis of severe TBI and GCS < 9 • Estimated age ≥ 18 and < 60 years • The patient is intubated or intubation is imminent
Pre-hospital exclusion criteria	Emergency Department exclusion criteria
• Clinical diagnosis of drug or alcohol intoxication as predominant cause of coma • Randomisation unable to be performed within 3 h of estimated time of injury • Estimated transport time to study hospital > 2.5 h • Able to be intubated without drugs • Systolic BP < 90 mmHg • Heart rate > 120 bpm • Cardiac arrest at the scene or in transit • GCS = 3 + un-reactive pupils • Penetrating neck/torso injury • Known or obvious pregnancy • Receiving hospital is not a study site • Evidence of current anti-coagulant treatment Known to be carer dependent due to a pre-existing neurological condition	• Clinical diagnosis of drug or alcohol intoxication as predominant cause of coma • Randomisation unable to be performed within 3 h of estimated time of injury • Able to be intubated without drugs • Persistent systolic BP < 90 mmHg • GCS = 3 + un-reactive pupils • Cardiac arrest at the scene or in transit • Clinically significant bleeding likely to require haemostatic intervention, for example: ○ Bleeding into the chest, abdomen or retro-peritoneum likely to require surgery ± embolisation ○ Pelvic fracture likely to require surgery ± embolisation ○ More than two long bone fractures requiring operative fixation • Penetrating neck/torso injury • Positive urine or blood pregnancy test • Evidence of current anti-coagulant treatment • Known to be carer dependent due to a pre-existing neurological condition • In the treating clinician’s opinion, ‘cooling’ is not in the patient’s best interest

### Randomisation

Randomisation 1:1 between trial hypothermia and normothermia was performed by paramedics or physicians using sequentially numbered, opaque, sealed envelopes (as used in the RICH [23] and RSI trials [24]), printed at the Australian trial coordinating office before distribution to sites. Envelopes were used because at the time of trial design it was not feasible to develop a centrally controlled, real-time electronic randomisation system across international emergency departments and pre-hospital ambulances services. The computer-generated randomised treatment allocation schedule (using Stata version 11 *ralloc* module http://fmwww.bc.edu/repec/bocode/r/ralloc.ado) [25] was stratified by hospital group or pre-hospital paramedic ambulance service with a permuted block scheme. Hospital strata were defined for participating hospitals in nine groups, comprising three Australian states (Victoria, Queensland, Western Australia), two localities in New Zealand (Auckland, Waikato) and four other countries (France, Switzerland, Saudi Arabia, Qatar). Stratification of pre-hospital randomisation schedules was also specified within the four networks where such enrolment was possible (Queensland, Victoria, Western Australia, France) (Table 2).

**Table 2:** The count of strata incorporated within the block-randomised 1:1 hypothermia / normothermia treatment allocation schedule

Pre-hospital	Stratum no.	Hospital region	Stratum no.
Yes^a^	1	Victoria^a^	5
Yes^a^	2	Western Australia^a^	6
Yes^a^	3	Queensland^a^	7
Yes^a^	4	France^a^	8
No		Switzerland	9
No		Saudi Arabia	10
No		Qatar	11
No		Auckland	12
No		Waikato	13

^a^Participating pre-hospital ambulance services and their relevant regional hospitals within each of these Australia states and France were each included as separate strata within the computerised generation of randomisation envelopes

Patients who fulfilled the inclusion criteria and had no exclusion criteria were randomised by opening the next available opaque envelope within the ambulance or hospital emergency room.

In the pre-hospital setting patients were assessed by study trained and affiliated ambulance paramedics and/or physicians. Patients who were enrolled in the study by a pre-hospital agency were assessed for study suitability and continuance on arrival in the first participating emergency department. Patients who were not enrolled in the study by a pre-hospital agency were assessed for study suitability on arrival in the associated hospital emergency department.

### Study objectives and endpoints

#### Primary outcome

The primary outcome will be each patient’s neurological outcome at six months from randomisation. This is defined from a standard mid-point dichotomisation of the eight level ordinal Extended Glasgow Outcome Scale (GOSE) [26] (Table 3), with favourable outcomes comprising lower moderate disability (GOSE 5) through upper good recovery (GOSE 8) and unfavourable outcomes comprising death (GOSE 1), the vegetative state (GOSE 2) or severe disability (GOSE 3 to 4).

**Table 3:** The eight-level ordinal Extended Glasgow Outcome Scale (GOSE)

Score	Description	Code
1	Dead	D
2	Vegetative state	VS
3	Lower severe disability	SD -
4	Upper severe disability	SD +
5	Lower moderate disability	MD -
6	Upper moderate disability	MD +
7	Lower good recovery	GR -
8	Upper good recovery	GR +

#### Secondary outcomes

Secondary outcome measures are:

- GOSE regarded as an ordinal variable (as opposed to the dichotomisation used as the primary outcome variable)
- Quality of life (QOL) assessments at six months:
  - ○ EQ5D3L [27]
  - ○ SF12 [28]
- Complier average causal effect of hypothermia on GOSE at six months comparing hypothermia and control patients
- Mortality (all cause) at:
  - ○ hospital discharge
  - ○ six months
- Proportion of patients with adverse events (AEs) within ten days of randomisation, specifically:
  - ○ bleeding (intracranial, extracranial)
  - ○ infection (by site)
- Cost-effectiveness at six months

### Statistical analyses

Descriptive and summary statistics will be calculated by treatment group and stratum (pre-hospital and emergency department region) for baseline characteristics. Continuous data will be summarised as means (standard deviations) or medians (interquartile ranges) for non-normal data and categorical data by counts and proportions. The number of screened patients who fulfilled study inclusion criteria and the number included in the primary and secondary analyses as well as all reasons for exclusions in primary and secondary analyses will be reported.

The main primary and secondary analyses will follow an ITT approach to define the full analysis patient set, based on all randomly assigned patients after the eight-patient run-in period, except those withdrawing consent for use of all trial data and those not fulfilling inclusion criteria and never receiving the intervention [20, 29].

The early intervention design of the POLAR study specifically anticipates incomplete adherence to assigned cooling treatment in the complex clinical context of the study cohort. It is expected that the main reason for incomplete compliance with cooling will be due to the effects of alcohol or other drugs confounding assessment of TBI severity at the time of randomisation, leading to inclusion of patients with impaired consciousness not due to severe TBI for whom ongoing cooling may be clinically unwarranted. Other anticipated reasons include extracranial trauma and coincident haemorrhage for which cooling may be at least temporarily undesirable in the context of a patient’s overall clinical condition.

#### Primary outcome

The primary outcome, the midpoint dichotomised GOSE, will be modelled as a binomial random variable, with a null hypothesis of equality between hypothermic and standard therapy groups in the proportion of subjects with an unfavourable outcome. This will be assessed with an uncorrected Chi-square test applied to the 2 × 2 contingency table comprising the full analysis set of patients according to randomised treatment group. This primary trial outcome will be reported as an unadjusted risk ratio with associated 95% confidence interval (CI) and also as the risk difference with 95% CI and odds ratio with 95% CI. The number needed to treat for benefit or harm will also be reported if a statistically significant difference between treatment groups is demonstrated.

#### Secondary outcomes

All secondary outcomes are hypothesis-generating. GOSE as an ordinal variable will be compared between treatment arms using a proportional odds model if the proportional odds assumption is justified [30, 31]. If not, a partial proportional odds model will be used [32, 33].

Binary variables (including hospital and six-month mortality and AEs) will be analysed with log-binomial [34] and identity-binomial regression models [35] to estimate risk ratios and risk differences with 95% CIs, respectively.

QOL measures and other continuous variables will be analysed with linear regression, using robust standard errors to accommodate potential non-normality and unequal error variation.

Analyses of time-to-event outcomes will use Kaplan–Meier plots and log-rank tests, as well as unadjusted and adjusted Cox proportional hazard regression models returning hazard ratios with 95% CIs.

QOL outcomes will be reported as means (with standard deviations) of the physical and mental health scores of the SF-12 [28] and as the proportion of reported health problems for each domain of the EQ5D-3 L [27]. Differences in QOL between groups will be assessed using two-sample t tests or Wilcoxon rank-sum tests as appropriate [20] for the SF-12 component scores and Fisher’s exact or Chi-squared tests for the proportions in each domain of the EQ-5D-3 L.

#### Cost-effectiveness analyses

Cost-effectiveness from the healthcare-payer perspective will be calculated as a cost per additional patient with a favourable neurological outcome at six months following randomisation (defined as GOSE 5–8) and the cost per additional quality-adjusted life year (QALY), with QALYs calculated using utility scores derived from the EQ-5D-3 L conducted at six months post randomisation. Costs will be determined based on resource use during the intensive care, acute and post-acute periods up to six months post randomisation. These will be valued using the UK time-trade-off tariff. All patients will be assumed to have a utility score of zero at randomisation in accordance with other studies recruiting patients in critical care. Patients who die before the six-month follow-up will be given a utility of zero.

Data on resource use at different levels of care will be recorded at discharge from the index hospitalisation and again at follow-up for subsequent healthcare resource use (including readmissions, rehabilitation and other care facilities). Information will be collected for length of ventilation, time in ICU, hospital (ward) days, rehabilitation days and time spent in high- and low-level care facilities and transitional living centres.

Resources will be translated into costs by multiplying the relevant country-specific unit cost by use for each patient in the analysis, where unit costs are obtained from hospital staff where possible, or national databases. All costs will be reported in 2017 prices using national consumer price index statistics from relevant countries. Purchasing power parity (PPP) statistics from the Organisation for Economic Co-operation and Development (OECD) will be used to translate costs to a common currency (United States Dollars, $US). Given the relatively short time period, costs will not be discounted.

Analyses will adjust for fixed effects across three geographical regions (Australia/New Zealand (ANZ), Europe and the Middle East) to account for potential heterogeneity which may arise, for example, through regional variations in treatment patterns. The issue of transferability in multinational trials will be addressed by estimating costs and effects for the same three geographical regions. Region-specific estimates will be obtained by interacting the treatment variable with regional fixed effects.

#### Pre-specified subgroup analyses

Two interactions of particular prior interest will be assessed. The primary and secondary outcomes will be evaluated according to: (1) the presence of surgically evacuated intracranial mass lesions (Marshall score V) [36]; and (2) the presence of any intracranial mass lesion whether or not surgically evacuated (Marshall V or VI).

#### Dose effect / Intensity of cooling

Intensity of cooling in intervention arm patients will be categorised according to the time after randomisation to first reach one of two core temperature thresholds, being the limit of mild hypothermia 35 °C [9] and also 34 °C as an indicator of more intense [6] cooling below 35 °C towards 33 °C. Cooling intensity categories are defined as never achieving hypothermia and tertiles of time in those reaching hypothermia. Primary and secondary outcomes of patients in these intensity categories will be compared across categories and to standard care patients.

Differential effects of cooling on the primary and secondary outcomes will be assessed according to: (1) Marshall computed tomography (CT) scan classification V; and (2) Marshall V or VI. These will be performed using appropriate covariate by treatment interaction terms in the relevant regression models.

#### Sensitivity analyses of the primary and secondary outcomes

Sensitivity analyses of the primary and secondary outcomes will be performed using regression models adjusting for pre-specified baseline covariates as well as any covariate exhibiting substantial imbalance between randomisation arms, as recommended [37]. Linear and generalised linear model diagnostics, outlier assessment and remedial measures will follow standard approaches [30, 38].

Proportionality in ordinal logistic regression models will be assessed [39]. Also, the proportional hazards assumption across treatment arms in time-to-event analyses will be evaluated using scaled Schoenfeld residuals [40] and visual assessment of log-log plots.

Baseline variables to be included as fixed effects when developing adjusted outcomes models comprise the following:

- Geographic region (Australia and New Zealand, Middle East, Europe – three level nominal categorical)
- Age (integer values centred on overall median age of trial patients)
- GCS (integer values with a possible range of 3–15)
- Pupil reactivity (ordinal, three levels)
- Hypoxia (binary)
- Hypotension (binary)
- Marshall CT brain scan classification (nominal categorical, six levels) [39]
- Core temperature closest to time of randomisation (continuous)

Generalised linear mixed regression models will be applied with the same fixed effects covariate set while also incorporating as a random effect the multiple randomisation strata comprising hospital and pre-hospital research groups. In the event the above regression models do not accommodate the number of pre-specified covariates, sensitivity analyses will be simplified to adjust at least for POLAR treatment and the baseline extended IMPACT TBI probability of 6 month unfavourable outcome.

#### Exploratory analyses

It is well understood that trial protocols may not have been followed fully for some trial participants [17]. The intervention under examination is hypothermia to a target of 33 °C core temperature initiated within 3 h of injury and continued for at least 72 h, with rewarming guided by an intracranial pressure < 20 mmHg. Beyond the main ITT analyses of the primary and secondary outcomes, pre-specified exploratory analyses will use ‘as-treated’ and ‘per-protocol’ comparison approaches as well as methods to estimate the ‘complier average causal effect’ (CACE) of cooling [22, 38].

##### As-treated and per-protocol analyses

POLAR will repeat the primary and secondary trial outcomes in two pre-defined exploratory analysis sets: (1) the subset of compliant patients assigned to cooling compared to all patients assigned to the control (‘per-protocol’ analysis); and (2) according to the actual treatment received (‘as-treated’ analysis) [7].

The per-protocol analysis subset will comprise control and intervention patients who are demonstrated retrospectively to have suffered a severe TBI, thus excluding those with initially low GCS < 9 not due to severe head injury but rather associated with transient influences, such as alcohol or drug effects.

For analytic purposes, the differential ‘dose’ of early cooling received by each patient will be summarised as the area (degree × hours) under the protocol-defined cooling baseline value of 35 °C within 72 h of randomisation. Each individual’s AUC will be estimated by the linear trapezoidal method [41] applied to temperature data ignoring missing values. Early death or loss to follow-up within 72 h of randomisation will by definition reduce the observed dose of cooling received by such patients. The intensity of cooling will also be summarised categorically, in increments of 1 °C from ≥ 38 °C to ≤ 32 °C, as the proportion of individuals whose lowest core temperature during the trial intervention days was in each of these temperature bands. A binary definition of ‘compliance with cooling’ will constitute an AUC at least 72 degree × hours, this being at least half that theoretically achievable in the first 72 h. This definition will be used to identify the per-protocol and as-treated populations.

##### Estimation of the average causal effect among patients complying with treatment

In trials with incomplete adherence to treatment, such as that anticipated in POLAR, the ITT comparison only provides an estimate of the causal effect of treatment assignment rather than an estimate of the causal effect of treatment actually received. CACE analyses will be conducted to estimate the average effect of cooling treatment on the primary outcome for patients who would comply with whichever cooling group they were assigned to, considering both the binary and continuous definitions of compliance with cooling [22, 38].

Estimation of the CACE will use an instrumental variables approach in the first instance, with a randomised arm as the instrument [42]. Alternatively, if compliance is a time-varying variable, estimation of the CACE will use longitudinal nested compliance class methods [43].

### Data monitoring

All numerical data fields in the trial database have upper and lower review limits based on biologically unlikely thresholds, so as to identify possible data entry errors. Also, all dates and times will be checked for logic errors. The POLAR Trial Manager monitored all research sites in person, with the assistance of a French-speaking trial monitor at the four sites in France. Monitoring visits checked multiple aspects of data validity, including consent documents, inclusion and exclusion criteria, AEs and important daily data such as temperature, use of vasoactive agents, fluid balance, blood transfusion and electrolyte disturbances (Additional file 3).

An independent Data and Safety Monitoring Committee (DSMC) continues to oversee the quality of the trial and has access to trial outcome and accumulated safety data, including the differential proportions of total mortality (Additional file 4).

### Sample size, power and interim analysis schedule

The overall recruitment target was set at 500 participants from commencement in December 2010 and was slightly increased to 510 participants in September 2017 after blinded review of the combined proportion of patients with ‘consent withdrawn’ or ‘loss to follow-up’. A total of 511 patients were randomised before the conclusion of trial recruitment on 10 November 2017.

A study of fixed size with full compliance and follow-up would require 364 patients (182 in each of two treatment arms) to detect a 15% absolute increase from 50% to 65% in favourable neurological outcome at six months following injury (equivalent to a 30% relative improvement in risk) with slightly > 80% (82.8%) power, assuming a two-sided alpha of 0.05 and an uncorrected Chi square test. This postulated POLAR risk improvement from a baseline of 50% to 65% in treated patients is equivalent to an odds ratio of approximately 1.86.

The original POLAR trial sample size of 364 fully evaluable patients was appropriately inflated to a practically required target of 500 patients to maintain 80% power to find the anticipated beneficial effect of prophylactic hypothermia while accommodating anticipated losses to follow-up (5%) and non-compliance (cross-over from cooling to control and related losses, maximum 12%). Also incorporated was a much smaller (0.7%) inflation necessary to accommodate the originally anticipated interim analyses of both mortality and the proportion of unfavourable neurological outcomes using Haybittle-Peto 3SD group sequential boundaries at two recruitment points (one-quarter [*n* = 125] and one-half [*n* = 250]) [7].

Following the October 2015 publication of the EUROTHERM3235 study [6], the POLAR DSMC required a further substantial increase in interim monitoring, namely at increments of 50 patients from *n* = 300 to *n* = 450 inclusive. Ten of 11 planned interim analyses will be of both mortality and the proportion of unfavourable neurological outcomes, while at the penultimate (*n* = 450) assessment, short-term 28-day mortality alone will be assessed due to time constraints. These extra analyses implied a sample size inflationary requirement (4% - 0.7% = 3.3% extra) [44] which may be accommodated within the originally planned sample size, provided losses to follow-up and non-compliance remained below anticipated limits.

From calculations using East trial design software [45] based upon an approximate Chi-square test, the trial power was only slightly diminished at 82.3% (down from 83%) with 366 fully evaluable participants and 11 interim Haybittle-Peto 3SD interim analyses. In the absence of early stopping, the final analysis would be properly conducted at ± 1.996 SD (*P* = 0.0459) rather than ± 1.96 SD (*P* = 0.05). This level of significance will not be adjusted for multiplicity; however, the primary trial outcome is clearly defined and the conclusions of the study will be those based on the primary analysis conducted in the ITT full analysis patient set. Unless otherwise specified, all hypothesis tests and accompanying significance levels (that is, *P* values) will be two-sided, with 95% CI.

## Analysis software

Data capture and processing occurs initially at Monash University Clinical Informatics and Data Management Unit [15] and these data will be exported in relevant formats for statistical analysis using current versions of SAS software [46] and Stata software [47] or similar statistical software.

## Safety and adverse event analyses

Safety and tolerability implications will be summarised using descriptive statistical methods, supplemented by calculation of 95% CI where appropriate. Patients with protocol deviations, AEs (Additional file 5: POLAR Data Dictionary Form12 Adverse Events) and missing values will be identified, and a descriptive analysis undertaken including their relationship to treatment.

## Discussion

Severe TBI is a common and devastating condition with few proven specific therapies available. The administration of early prophylactic hypothermia has the potential to reduce neurological damage and improve neurological outcome and is supported by a scientific rationale and laboratory data. The POLAR-RCT is designed to detect an important beneficial effect of early therapeutic hypothermia on neurological function if one exists, while minimising any potential risks after TBI with accompanying illness or extracranial injury. Application of this statistical analysis plan to the POLAR-RCT trial will facilitate evaluation of these important clinical data and support confidence in the subsequent generalisation of its findings. POLAR-RCT aims to provide definitive guidance for clinicians regarding the true efficacy and safety of early prophylactic hypothermia in the management of TBI.

## Trial status

The POLAR-RCT began in December 2010, with patient randomisation completed on 10 November 2017 and final collection of all six-month outcome data anticipated by June 2018. A published manuscript describes the details of the trial protocol [7]. The current protocol is version 9, dated 11 July 2017.

## Additional files

Additional file 1: POLAR-RCT outcome assessors. (DOCX 28 kb)

Additional file 2: SPIRIT 2013 Checklist. (DOC 141 kb)

Additional file 3: Study Administration Structure. (DOCX 34 kb)

Additional file 4: POLAR-RCT Medical Monitor and Data Safety Monitoring Committee. (DOCX 29 kb)

Additional file 5: POLAR Data Dictionary Form12 Adverse Events. (DOCX 45 kb)

Additional file 6: POLAR-RCT Management Committee and Coordinating Centre. (DOCX 29 kb)

Additional file 7: The POLAR Study Investigators. (DOCX 31 kb)

Additional file 8: Responsible Ethics Committees. (DOCX 30 kb)

## Abbreviations

ANZIC-RC: Australian and New Zealand Intensive Care Research Centre
AUC: Area under curve
CI: Confidence interval
DSMC: Data and Safety Monitoring Committee
EQ-5D: EuroQol five dimensions
GCS: Glasgow Coma Score
GOSE: Extended Glasgow Outcome Scale
SF-12: Short Form-12
TBI: Traumatic brain injury
