# Supplementary material for: Statistical analysis plan for the POLAR-RCT: The Prophylactic hypOthermia trial to Lessen trAumatic bRain injury-Randomised Controlled Trial
Source: Trials. 2018 Apr 27;19:259. doi: 10.1186/s13063-018-2610-y (PMC5923032; doi:10.1186/s13063-018-2610-y)
Supplement: Supplementary file 1 — POLAR-RCT outcome assessors. (DOCX 28 kb) [file 13063_2018_2610_MOESM1_ESM.docx]

# Additional file 1: POLAR-RCT outcome assessors

Dr. Heather Waddy, Australian and New Zealand Intensive Care Research Centre, Monash University, Melbourne, Australia

Dr. Elisabeth De Bustos Medeiros, Department of Neurology, CHRU de Besançon, Hôpital Jean Minjoz

Ms Nicole Söll, Department of Neurosurgery, Inselspital, Bern University Hospital, University of Bern, Bern, Switzerland

Lynnette Murray, Australian and New Zealand Intensive Care Research Centre, Monash University, Melbourne, Australia

Dr. Dashiell Gantner, Medical Monitor, Intensive Care Unit, The Alfred Hospital, Melbourne, Victoria, Australia and Australian and New Zealand Intensive Care Research Centre, School of Public Health and Preventive Medicine, Monash University, Melbourne, Australia

Ms Victoria Bennett, Assistant to Medical Monitor, Australian and New Zealand Intensive Care Research Centre, School of Public Health and Preventive Medicine, Monash University, Melbourne, Australia
