# Supplementary material for: Statistical analysis plan for the POLAR-RCT: The Prophylactic hypOthermia trial to Lessen trAumatic bRain injury-Randomised Controlled Trial
Source: Trials. 2018 Apr 27;19:259. doi: 10.1186/s13063-018-2610-y (PMC5923032; doi:10.1186/s13063-018-2610-y)
Supplement: Supplementary file 3 — Study Administration Structure. (DOCX 34 kb) [file 13063_2018_2610_MOESM3_ESM.docx]

# Additional file 3: Study Administration Structure


### Coordinating Centre and Data Management Centre

Australian and New Zealand Intensive Care Research Centre (ANZIC-RC) and Department of Epidemiology and Preventive Medicine (DEPM) Monash University, Victoria, Australia

#### Responsibilities

- Overall management of the study including assistance with HREC applications
- Management of study budget and liaison with funding bodies
- Protocol and case report form (CRF) design and production
- Database design and management
- Protocol training of research coordinators and POLAR study team
- Protocol training of ambulance paramedics
- Preparation and arrangement of investigator payments
- Study set-up
- Randomisation
- Coordination of data entry and feedback of data enquiries
- Monitoring and close-out site visits
- Organisation of investigator meetings
- Serious adverse event notification
- Data analysis and collaboration on publications

#### Staff

| 1. Prof Jamie Cooper | Principal Investigator, ANZIC-RC |
| --- | --- |
| 1. Ms Lynne Murray | Research Manager, ANZIC-RC |
| 1. Prof Alistair Nichol | Professor, ANZIC-RC |
| 1. Prof Andrew Forbes | Head Biostatistics Unit, DEPM, Monash University |
| 1. Mr Tony Trapani | Project Manager, ANZIC-RC |
| 1. Prof Michael Bailey | Biostatistician, ANZIC-RC |

#### Meetings

As required.

### Executive Committee

#### Responsibilities

- Day to Day study management
- Liaise with Management Committee and sites

#### Members

| Professor Jamie Cooper | Principal investigator ANZIC-RC |
| --- | --- |
| Professor Alistair Nichol | Professor, ANZIC-RC |
| Professor Stephen Bernard | Medical Advisor, Ambulance Victoria |
| Mr Tony Trapani | Project Manager, ANZIC-RC |
| Ms Lynne Murray | Research Manager, ANZIC-RC |

#### Meetings

Weekly and as required

### Management Committee

#### Responsibilities

Overseeing all aspects of the study management including:

- Liaison with coordinating centre staff
- Liaison with steering committee Liaison with ambulance services
- Overseeing funding applications
- Overseeing disbursement and administration of funds
- Ensuring fiscal responsibilities are maintained
- Development and approval of final protocol and study materials
- Development and approval of data collection tools and methods
- General study management issues
- Liaison with ANZICS CTG

| Professor Stephen Bernard | Medical Advisor, Ambulance Victoria |
| --- | --- |
| Professor Jamie Cooper | Principal investigator ANZIC-RC |
| Professor Peter Cameron | Head of Emergency Trauma Research Group, DEPM, Monash University |
| Professor Andrew Forbes | Head Biostatistics Unit, DEPM, Monash University |
| Dr Colin McArthur | Director Critical Care Medicine, Auckland City Hospital |
| Ms Lynne Murray | Research Manager, ANZIC-RC |
| Ms Lynette Newby | Research Coordinator, DCCM, Auckland City Hospital |
| Professor Alistair Nichol | Senior Lecturer, ANZIC-RC |
| A/ Professor Jeffrey Presneill | Staff Specialist, Intensive Care Unit, Royal Melbourne Hospital |
| Dr Stephen Rashford | Medical Director, Queensland Ambulance Service |
| Professor Jeffrey Rosenfeld | Neurosurgeon, The Alfred |
| Dr Tony Smith | Medical Advisor, St John Northern Region, NZ Ambulance Service |
| Mr Michael Stephenson | MICA Group Manager, Ambulance Victoria |
| Mr Tony Trapani  Ms Shirley Vallance | Project Manager, ANZIC-RC  Research Co-Ordinator, The Alfred Hospital ICU |
| A/Professor Tony Walker | General Manager Quality and Education Services, Ambulance Victoria |
| Professor Steve Webb | Intensive Care Consultant, Royal Perth Hospital |
| Professor Capellier | Critical Care and Emergency Department, Besançon, France |
| Assoc/Prof Dinesh Varma | Deputy Director of Radiology, The Alfred, Melbourne |

#### Meetings

4-6 weekly

### Steering Committee

#### Responsibilities

- Oversight and advisory role
- Data analysis, collaboration and approval of study publications

#### Members

- Management committee (as above)
- State/Country Ambulance Service investigators
- Associate investigators*

*Local representatives appointed once sites confirmed (suggested 1 Intensive Care Physician, 1 Neurosurgeon, 1 Emergency Dept Physician, 1 Research Coordinator per site)

#### Meetings

As required
