# Supplementary material for: Statistical analysis plan for the POLAR-RCT: The Prophylactic hypOthermia trial to Lessen trAumatic bRain injury-Randomised Controlled Trial
Source: Trials. 2018 Apr 27;19:259. doi: 10.1186/s13063-018-2610-y (PMC5923032; doi:10.1186/s13063-018-2610-y)
Supplement: Supplementary file 4 — POLAR-RCT Medical Monitor and Data Safety Monitoring Committee. (DOCX 29 kb) [file 13063_2018_2610_MOESM4_ESM.docx]

# Additional file 4: POLAR-RCT Medical Monitor and Data Safety Monitoring Committee

**Data Safety Monitoring Committee**

| **DSMC Chair:** | **Assoc/Prof Jamie Hutchison**  Research Director Critical Care Medicine, The Hospital for Sick Kids, Toronto, Ontario, Canada |
| --- | --- |
| **DSMC Members**: | **Prof Paul Hébert**  Intensivist, Ottawa Hospital, Ottawa, Ontario, Canada |
|  | **Dr David Zygun**  Assistant Professor, University of Calgary, Calgary, Alberta, Canada |
|  | **Dr Alexis Turgeon**  Assistant Professor, Research Director, Division of Critical Care Medicine, Dept of Anaesthesiology, Universite Laval, Quebec, Canada |
|  | **Assoc/Prof Dean Fergusson**  Director, Methods Centre, Ottawa Medical Health Institute, Ottawa, Canada. |
| **Statistician** | **Prof Michael Bailey**  Senior Statistical Consultant, ANZIC RC, Monash University |
