# Supplementary material for: Statistical analysis plan for the POLAR-RCT: The Prophylactic hypOthermia trial to Lessen trAumatic bRain injury-Randomised Controlled Trial
Source: Trials. 2018 Apr 27;19:259. doi: 10.1186/s13063-018-2610-y (PMC5923032; doi:10.1186/s13063-018-2610-y)
Supplement: Supplementary file 5 — POLAR Data Dictionary Form12 Adverse Events. (DOCX 45 kb) [file 13063_2018_2610_MOESM5_ESM.docx]

| Form 12 --- Adverse Events | | | |
| --- | --- | --- | --- |
| **Notes:**  **Complete this form for all patients with an adverse incident.**  Patient Study Number and initials will be auto-filled.  **The POLAR study has predetermined Adverse Events. If an AE occurs that is not listed it can be documented in 12.16**  **Some Adverse Events will also require** Serious Adverse event form to be completed. | | | |
| 12.1 | Did an SAE occur? | **Some Adverse Events will also require** Serious Adverse event form to be completed  **Consider the seriousness of the adverse event and whether it will require an SAE form to be completed.**  **Answer “YES” or “NO”** |  |
| *Adverse Events (1 of 4) - Cardiac*  **Guidelines**  **An Adverse Event (Cardiac) will be documented if it occurs:**  **During the ICU admission and/or up to 10 days from randomisation**  *The CRF should be marked ‘YES’ if it is the opinion of the principal investigator or the consultant responsible for the patient on that day, there was probable or proven new adverse event.* | | |  |
| 12.2.1 | Bradycardia | **Heart rate < 40** and/or associated with haemodynamic compromise (MAP < 60mmHg) / or increase use of a vasopressor. |  |
| 12.2.2 | Number of events | During the ICU admission and/or up to 10 days from randomisation |  |
| 12.3.1 | Ventricular Tachycardia | **Three (3) or more consecutive ventricular beats** at a ventricular rate of greater than 100**.**  With or without haemodynamic compromise. |  |
| 12.3.2 | Number of events | During the ICU admission and/or up to 10 days from randomisation |  |
| 12.4.1 | Ventricular Fibrillation | **Chaotic ventricular activity** confirmed on a rhythm strip.  . |  |
| 12.4.2 | Number of events | During the ICU admission and/or up to 10 days from randomisation |  |
| 12.5.1 | Asystolic or PEA arrest | **No cardiac electrical activity** or **electrical activity with NO arterial blood pressure** |  |
| 12.5.2 | Number of events | During the ICU admission and/or up to 10 days from randomisation |  |

| *Adverse Event (2 of 4) - Hypotension*  **Guidelines**  **An Adverse Event (Hypotension) will be documented if it occurs**  **Within the period prescribed below.**  *The CRF should be marked ‘YES’ if it is the opinion of the principal investigator or the consultant responsible for the patient on that day, that there was probable or proven new adverse event.* | | |
| --- | --- | --- |
| 12.6.1 | MAP<60mmHg | Did the patient’s **Mean Arterial Blood Pressure fall** below 60mmHg  Answer **YES** or **NO.**  **If answer is YES continue to next question and document when Hypotension occurred.** |
| 12.6.2 | Induction of Cooling | Did the drop in MAP (12.6.1) occur during **induction of cooling?**  (Induction is from the time of randomisation to time the patient reaches 33^o^C.)  How many times **during the induction of cooling phase** did the patient’s **MAP drop below 60mmHg**?  If **Mean Arterial Blood Pressure did NOT fall** below 60mmHg enter “0” |
| 12.6.3 | Maintenance of Cooling | Did the drop in MAP (12.6.1) occur during **maintenance of cooling?**  (Maintenance is from time the patient reaches 33^o^C until rewarming commences)  How many times **during the maintenance of cooling** **phase** did the patient’s **MAP drop below 60mmHg**?  If **Mean Arterial Blood Pressure did NOT fall** below 60mmHg enter “0” |
| 12.6.4 | Rewarming | Did the drop in MAP (12.6.1) occur during **re-warming phase?**  Rewarming is from time the patient commences to rewarm from 33^o^C to the time the patient reaches 36.5+/-0.5^o^C  How many times **during the re-warming phase** did the patient’s **MAP drop below 60mmHg**?  If **Mean Arterial Blood Pressure did NOT fall** below 60mmHg enter “0” |
| 12.6.5 | Normothermia | Did the drop in MAP (12.6.1) occur while the patient **was normothermic?**  Normothermia is defined as completion of rewarming and up to ICU discharge or 10 days post randomisation.  OR  In the “normothermic study group” this is the period from randomisation up to ICU discharge or 10 days post randomisation.  How many times **during the normothermia phase** did the patient’s **MAP drop below 60mmHg**?  If **Mean Arterial Blood Pressure did NOT fall** below 60mmHg enter “0” |

| *Adverse Event (3 of 4) – Infection*  **Guidelines**  An Adverse Event (Infection) will be documented if it occurs  **During the ICU admission and/or up to 10 days from randomisation**  *The CRF should be marked ‘YES’ if it is the opinion of the principal investigator or the consultant responsible for the patient on that day, that there was probable or proven new adverse event.*  *In each situation the finding of a new organism would be counted as a new infection.* | | |
| --- | --- | --- |
| 12.7.1 | Pneumonia | Physician considers the patient has a pneumonia and is treating for same.  *Supporting evidence (not mandatory):*  Radiographic infiltrate  Clinical Suspicion (which may include the following)   - Increase WCC (>12K or <4K) - Worsening oxygenation - Isolation of a likely pulmonary pathogen - Purulent sputum |
| 12.7.2 | Number of events | During the ICU admission and/or up to 10 days from randomisation |
| 12.8.1 | Proven Bacteremia | Physician considers the patient has a bacteraemia and is treating for same.  *Supporting evidence (not mandatory):*  New antibiotic commenced  Recognized pathogen cultured from one or more blood cultures (or in common skin contaminant, is cultured from 2 or more blood cultures).  ***Each new contaminant would be a new bacteraemia*** |
| 12.8.2 | Number of events | During the ICU admission and/or up to 10 days from randomisation |
| 12.9.1 | Cerebral Abscess | Physician/surgeon considers the patient has a Cerebral Abscess and is treating same. |
| 12.9.2 | Number of events | During the ICU admission and/or up to 10 days from randomisation |
| 12.10.1 | CNS Infection | Physician/surgeon considers the patient has a CNS infection (ventriculitis or meningitis) and is treating same.  *Supporting evidence (not mandatory):*   - Patient has organism cultured from Cerebrospinal Fluid - Increase WCC / elevated Protein and or decreased glucose in CSF - Organisms seen on gram stain |
| 12.10.2 | Number of events | During the ICU admission and/or up to 10 days from randomisation |
| 12.11.1 | Neurosurgical Wound Infection | Physician/surgeon considers the patient has a neurosurgical wound Infection and is treating same.  *Supporting evidence (not mandatory):*   - Purulent discharge from wound - Signs of erythema, blanching, tenderness, pain, purulent discharge, heat |
| 12.11.2 | Neurosurgical wound Infection | During the ICU admission and/or up to 10 days from randomisation |
| 12.12.1 | Other Infection | Physician/surgeon considers the patient has an infection.  Please specify  *Supporting evidence (not mandatory):*  Identification via laboratory results or via radiological or nuclear scanning. |
| 12.12.2 | Number of events | During the ICU admission and/or up to 10 days from randomisation |

| *Adverse Event (4 of 4) – Bleeding*  **Guidelines**  An Adverse Event (Bleeding) will be documented if it occurs  **During the ICU admission and/or up to 10 days from randomisation**  *The CRF should be marked ‘YES’ if it is the opinion of the principal investigator or the consultant responsible for the patient on that day, that there was probable or proven new adverse event.* | | |
| --- | --- | --- |
| 12.13.1 | New Intracerebral Bleed | A new bleed intra cerebral haematoma on CT /MRI or documented intraoperatively.  Not following the expected course of injury. |
| 12.13.2 | Number of events | During the ICU admission and/or up to 10 days from randomisation |
| 12.14.1 | New significant bleeding | A decrease in the haemoglobin concentration of at least 2 g/dL in 24 hours and the transfusion of 2 units of packed red cells within 24 hours after bleeding  Failure of the haemoglobin concentration (in g/dL) to increase after transfusion by at least the number of units transfused minus 2 (i.e. if 4 units of packed cells were transfused, the bleeding would be considered clinically important if the haemoglobin concentration did not rise by at least 2 g/dL |
| 12.14.2 | Number of events | During the ICU admission and/or up to 10 days from randomisation |
| *Intractable Hypertension* | | |
| 12.15.1 | Intractable ICP | How many events did the patient have an (untreatable and sustained) ICP >20? An event is not the number of hours.   - **ICP >20mmHG** - **and sustained continuously at this level for >20min for some >1 hour** - **Patient on maximal therapy or required additional therapy or dramatic increase in therapy** |
| 12.15.2 | Number of Events | During the ICU admission and/or up to 10 days from randomisation |
| *Intractable Hypertension* | | |
| 12.16 | Increase in Intracerebral Bleed | A new bleed or a significant extension of a previous intra cerebral haematoma on CT /MRI or documented intraoperatively.  Not following the expected course of injury.  **Please ensure that De identified CT report is sent to Project Manager** |
| *Other adverse events* | | |
| 12.17 | List | **Please write comment about Adverse Incident** |
