# Supplementary material for: Statistical analysis plan for the POLAR-RCT: The Prophylactic hypOthermia trial to Lessen trAumatic bRain injury-Randomised Controlled Trial
Source: Trials. 2018 Apr 27;19:259. doi: 10.1186/s13063-018-2610-y (PMC5923032; doi:10.1186/s13063-018-2610-y)
Supplement: Supplementary file 6 — POLAR-RCT Management Committee and Coordinating Centre. (DOCX 29 kb) [file 13063_2018_2610_MOESM6_ESM.docx]

# Additional file 6: POLAR-RCT Management Committee and coordinating Centre.

**Chief Investigator:** Professor Jamie Cooper

Director Research, Intensive Care Unit, The Alfred

Melbourne, Victoria 3004, Australia

**Management Committee:** Professor Stephen Bernard

Professor Peter Cameron

Professor Gilles Capellier

Professor Andrew Forbes

Dr Colin McArthur

Ms Lynne Murray

Professor Alistair Nichol

Ms Lynette Newby

Assoc/Professor Jeffrey Presneill

Dr Stephen Rashford

Professor Jeffrey Rosenfeld

Dr Tony Smith

Mr Michael Stephenson

Mr Tony Trapani

Ms Shirley Vallance

Assoc/Professor Tony Walker

Professor Steve Webb

Assoc/Professor Dinesh Varma

**Coordinating Centre:**  Australian and New Zealand Intensive Care Research Centre

Department of Epidemiology and Preventive Medicine

School of Public Health and Preventive Medicine, Monash University

The Alfred Centre, 99 Commercial Road

Melbourne, Victoria 3004, Australia

<mailto:anzicrc@monash.edu>
