# Supplementary material for: Statistical analysis plan for the POLAR-RCT: The Prophylactic hypOthermia trial to Lessen trAumatic bRain injury-Randomised Controlled Trial
Source: Trials. 2018 Apr 27;19:259. doi: 10.1186/s13063-018-2610-y (PMC5923032; doi:10.1186/s13063-018-2610-y)
Supplement: Supplementary file 7 — The POLAR Study Investigators. (DOCX 31 kb) [file 13063_2018_2610_MOESM7_ESM.docx]

# Additional file 7: The POLAR Study Investigators.

**The Alfred Hospital, Melbourne, Australia:** Prof DJ Cooper, Prof S Bernard, Dr O Roodenberg, Prof JV Rosenfeld , Prof P Cameron , A/Prof A Udy, Mrs S Vallance , Ms J Broad

**The Royal Melbourne Hospital, Melbourne, Australia:** A/Prof C MacIsaac, A/Prof N Harley, A/Prof J Presneill, Dr T Rechnitzer, Dr S Sriram, Dr R D’Costa, Dr C Karcher, Dr K Gorman, Dr J Knott, Prof R Judson , Ms D Barge

**Princess Alexandra Hospital, Brisbane, Australia:** A/Prof C Joyce, Dr L Nunnink, Dr H Fuentes, Dr E Burkett, Dr J Walsham, Dr G Livesay, Mr K Perkins , Miss E Saylor , Miss E Venz , Mr J Meyer

**Gold Coast University Hospital, Gold Coast, Australia:** Prof M Wullschleger, Dr. J Winearls, Dr. B Richards, Ms. M Tallott, Ms. E Wake ,

**Royal Perth Hospital, Perth, Australia:** Prof S Webb , Dr E Litton, Dr S Honeybul, Dr N Henry, Prof D Fatovich, Prof S Brown, S Waterson

**Auckland City Hospital, Auckland, New Zealand:** A/Prof C McArthur , Dr T Smith , Ms L Newby

**Waikato Hospital, Hamilton, New Zealand:** Dr Robert Frengley, Mr J Durning, Mrs Mary LaPine

**CHRU Besançon Hôpital Jean Minjoz, Besançon, France:** Prof G Capellier , Prof S Pili-Floury, Ms L Vetteroti VETTORETTI, Ms. Gaelle Amiotte

**CHU de Clermont Ferrand, Hôpital Gabriel Montpied, Clermont Ferrand, France:** Dr Russell Chabanne, Elodie Caumon

**Hôpitaux Universitaires de Strasbourg, Hôpital de Hautepierre, Strasbourg, France:** Prof. J Pottecher, Ms. S Hecketsweiler

**CHRU de Brest, Hôpital de la Cavale Blanche, Brest France:** Prof Olivier Huet, Dr Veronique Vermeersch, Dr David Goetghebeur, Dr Olivier Grimault, Ms Patricia Dias

**CHU de Nimes, Hopital Carémeau, Nimes, France :** Prof Jean Yves LeFrant, Ms. L Elotmani

**Department of Intensive Care Medicine, Inselspital, Bern University Hospital, University of Bern, Bern, Switzerland:** Dr. M Haenggi, Ms. M Roth

**King Abdulaziz Medical City, Riyadh, Saudi Arabia**: A/Prof S Alsolamy, Prof Y Arabi, Mr A Deeb , Ms N Al Assmi , Ms H Anizi

**Hamad General Hospital, Doha, Qatar:** Dr Ayman Ahmed El-Menyar, Dr. Hassan Al-Thani

**Pre-Hospital organisations**

**St John Ambulance (Western Australia):** Prof Ian Jacobs

**Ambulance Victoria:** A/Prof T Walker, Mr M Stephenson

**Queensland Ambulance Service:** Dr S Rashford , Dr. D Bodnar, Mr. L Parker

**SAMU-SMUR 25:** Prof G Capellier, Dr L Fehner, Mr A Journot

**SAMU-SMUR 29:** Dr Olivier Grimault

**SAMU-SMUR 63:** Dr F Dissait

**SAMU-SMUR 67:** Dr L Tritsch, Dr H Arzouq
