# Supplementary material for: Statistical analysis plan for the POLAR-RCT: The Prophylactic hypOthermia trial to Lessen trAumatic bRain injury-Randomised Controlled Trial
Source: Trials. 2018 Apr 27;19:259. doi: 10.1186/s13063-018-2610-y (PMC5923032; doi:10.1186/s13063-018-2610-y)
Supplement: Supplementary file 8 — Responsible Ethics Committees. (DOCX 30 kb) [file 13063_2018_2610_MOESM8_ESM.docx]

# Additional file 8: Responsible Ethics Committees

|  | ***Site Name*** | ***Ethics reviewing committee*** |
| --- | --- | --- |
| ***Australia*** | Royal Perth Hospital | Government of Western Australia, Department of Health, South Metropolitan Health Service HREC |
|  | Royal Melbourne Hospital | Melbourne Health, Human research Ethics Committee |
|  | The Alfred | Alfred Hospital Ethics Committee |
|  | Auckland DCCM | Northern A Health and Disability Ethics Committee |
|  | Waikato | Northern X Health and Disability Ethics Committee |
|  | Princess Alexandra Hospital | Metro South Hospital and Health Service, Human research Ethics Committee |
|  | Gold Coast University Hospital | Metro South Hospital and Health Service, Human research Ethics Committee |
|  | Ambulance Victoria | Ambulance Victoria, Research & Evaluation Department |
|  | Queensland Ambulance Service | Department of Health , Queensland Ambulance Service, Office of the Commissioner, Information Support, Research & Evaluation |
|  | St John's Ambulance Helicopter service | The University of Western Australia, Human Research Ethics committee |
|  | Monash University | Monash University Human Research Ethics Committee |
| ***New Zealand*** | Auckland DCCM | Northern A Health and Disability Ethics Committee |
|  | Waikato | Northern X Health and Disability Ethics Committee |
| ***France*** | CHRU Besançon - hôpital St Jacques | Comite de Protection des Personnes |
|  | CHU Clermont-Ferrand - Hôpital Gabriel Montpied | Comite de Protection des Personnes |
|  | Hôpitaux Universitaires de Strasbourg, Hôpital de Hautepierre | Comite de Protection des Personnes |
|  | CHRU Brest - Hôpital La Cavale Blanche | Comite de Protection des Personnes |
|  | CHU de Nimes, Hopital Carémeau, Nimes, France | Comite de Protection des Personnes |
| ***Switzerland*** | Bern University Hospital | Swiss Ethics Committee on research involving humans, Kantonale Ethikkommission, Bern |
| ***Kingdom of Saudi Arabia*** | King Abdullah International Medical Research Center | Ministry of National Guard Health Affairs, King Abdulah International Medical Research Center, Institutional review Board, Biomedical Ethics section |
| ***Qatar*** | Hamad General Hospital | Hamad Medical Corporation, Institutional review Board |
